# Improvement in appetite among stunted children receiving nutritional intervention in Bangladesh: results from a community-based study

**DOI:** 10.1038/s41430-020-00843-9

**Published:** 2021-05-27

**Authors:** Nurun Nahar Naila, Mustafa Mahfuz, Muttaquina Hossain, Michael Arndt, Judd L. Walson, Baitun Nahar, Tahmeed Ahmed

**Affiliations:** 1 Nutrition and Clinical Services Division (NCSD), International Centre for Diarrhoeal Disease Research, Bangladesh (icddr, b), Dhaka, Bangladesh; 2 PATH, Seattle, WA USA; 3 Department of Global Health, University of Washington, Seattle, WA USA; 4 Childhood Acute Illness and Nutrition Network, Nairobi, Kenya; 5 James P. Grant School of Public Health, BRAC University, Mohakhali, Dhaka 1212 Bangladesh

**Keywords:** Diseases, Nutrition disorders, Malnutrition

## Abstract

**Background/objectives:**

Stunted children often have poor appetite, which may limit their response to nutritional interventions. We investigated the effect of a nutritional intervention on the appetite status of stunted children.

**Methods:**

A longitudinal prospective intervention study was conducted with 50 stunted (length for age; LAZ < −2) (age and sex matched) aged 12–18 months and their mothers in Bauniabadh slum of Dhaka city. The stunted children received the following intervention package: one boiled egg and 150 ml milk daily 6 days a week for 3 months; psychosocial stimulation including structured play activities and parental counseling for 6 months; routine clinical care. Appetite status was measured using an interview-based tool “Early Childhood Appetite and Satiety Tool.”

**Results:**

Over the period of nutritional intervention, the mean appetite score increased from 49 to 60 in the stunted children and was associated with increased food consumption. Over the intervention period, both egg and milk consumption increased (40.3–49.6 g and 83.8–138.5 ml, respectively).

**Conclusions:**

Assessment of appetite status using EACST appears to be a useful tool for monitoring a nutritional intervention in stunted children. This tool may be useful for programs in managing child stunting in low-income countries and an important way to assess the efficacy of a nutritional intervention in these children.

## Introduction

Stunting (length-for-age *z*-score, LAZ < −2SD) in children represents chronic undernutrition and is associated with poor health outcomes, decreased learning capacity, and diminished productivity [1–3]. Stunting is now more prevalent than other forms of undernutrition, such as wasting and underweight, affecting an estimated 154.8 million children aged < 5 years in developing countries [4, 5]. Many of the effects of stunting appear to be nearly irreversible after 3–4 years of age [6].

The majority of the world’s stunted children reside in Africa and Asia, where 87 and 59% of the children under 5 years are stunted, respectively [4]. In Bangladesh, an estimated 31% of under 5 children are stunted and 9% are severely stunted (HAZ/LAZ < −3SD) [7]. The causes of stunting are multi-sectoral and multifactorial. Major determinants include intrauterine growth restriction, low birth weight, low maternal height, food insecurity, and inappropriate infant and young child feeding practices. Recurrent infections, micronutrient deficiencies, environmental enteric dysfunction, nonresponsive feeding practices, inadequate child stimulation at home [8], and lack of appetite [9] also have a role in occurrence of stunting [10, 11]. Linear growth faltering may be potentiated directly by these factors or through the mediation of decreased appetite [12]. It is evident that current interventions to reverse growth failure and improve health and cognitive outcomes are minimally effective in stunted children.

Nutritional interventions do play an important role in reversing stunting [2, 13]. Unfortunately, single interventions like food or micronutrient supplementation do not appear to effectively normalize growth and reduce morbidity in stunted children [9, 14, 15]. However, data suggest that existing nutritional interventions can reduce stunting prevalence by ~20% in children under 2 years of age and with all known effective interventions, approximately a third of all stunting could be averted. However, in order to achieve this impact, coverage of feeding interventions, including appropriate complementary feeding and other nutritional approaches, would need to reach ~99% [6]. One key factor which limits the impact of nutrition interventions may be the inability of stunted children to increase their dietary intake to meet increased metabolic demands [9, 14–18]. Lack of appetite may be a significant barrier to effective nutritional rehabilitation in these children.

Previous longitudinal studies in developed countries have demonstrated considerable improvement in nutritional status through community-based management, such as food and micronutrient supplementations, medical care and growth monitoring [19–21]. In addition to improvements in weight and length/height gain, these studies have also reported decreases in mortality [22–24]. In addition to nutritional interventions, psychosocial stimulation (PS) also has been shown to substantially contribute to the improvement of nutritional status in malnourished children [25, 26], although most of these studies have included only moderate and severely wasted hospitalized children [16, 26, 27].

The majority of studies evaluating nutritional interventions in malnourished children have assessed children’s appetite based on the amount of food they consume [12, 17, 18] rather than using a robust and dedicated measure of appetite status. Such measures are difficult to collect and often rely on caregiver report, which is subject to a number of sources of bias. Therefore, measuring appetite status in children [28] may be a useful tool to monitor the effectiveness of nutrition intervention. Accurate appetite measurements may improve the ability of programs to effectively manage and treat malnutrition in low-income countries, particularly stunting. Recently, we developed and validated a new tool to measure appetite status of under-five children in Bangladesh [28]. The aim of the present study is to determine whether appetite status improves in stunted children who receive a combined intervention (food supplementation (FS) and PS) and living in a slum of Dhaka city.

## Methods

### Study design

This is a community-based longitudinal prospective study.

### Sample size calculation

The sample size was calculated based on FGF-21 using estimates from a previous study [29]. In that study FGF-21 was found correlated positively with percent trunk fat (*r* = 0.25, *P* = 0.001) and bone mineral density (*r* = 0.30, *P* < 0.0001). We assumed that the correlation between FGF-21 level and nutrition intervention in stunted children (group 1) would differ by 0.6 from non-stunted children (group 2).

On the basis of the information, the sample size is 36 children per group assuming 80% power with level of significance, *α* = 0.05.

Sample size *N* = 36

Attrition rate 30% = 36 × 0.3 = 10.8 (11 rounded)

Total sample size per group = 36 + 11 = 47 (50 rounded)

Therefore, the total sample size for was 100 children of age 12–18 months.

### Study site and participants

In group 1 (stunted/intervention children), 50 stunted (LAZ < −2) children aged 12–18 months and their mothers, living in Bauniabad slum of Mirpur, Dhaka city, were enrolled after getting written informed consent from their caregivers. Children with severe acute malnutrition (weight-for-length *z*-score < −3 with or without edema), chronic illnesses, congenital anomalies, or developmental delay were excluded.

**Fig. 1:**
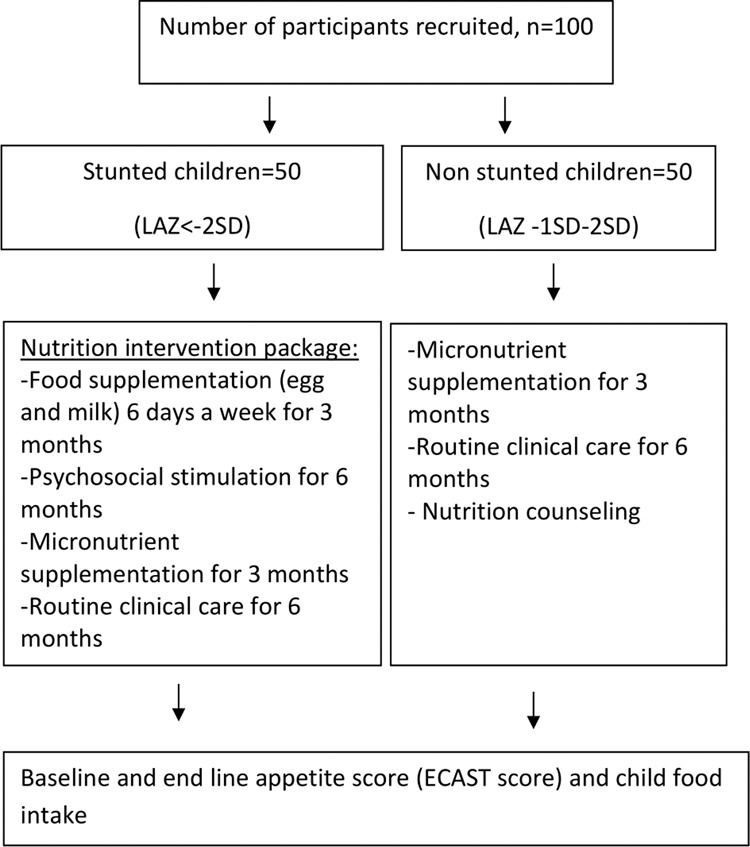
Study design. We recruited 50 stunted and 50 non-stunted children aged between 12–18 months whose length-for-age Z-score was less than −2 and morethan or equal to −2 respectively. The enrolled stunted children received an intervention package on site, which included food supplementation (FS), micronutrient supplementation (MNP), psychosocial stimulation (PS) and routine clinical care. The non-stunted children received routine clinical care, MNP, and nutritional counseling but no FS or PS. At the end of intervention baseline and end line appetite score and food intake were measured.

In group 2 (non-stunted/control children), 50 non-stunted (LAZ ≥ −2) children and their caregivers were also enrolled to examine if changes in appetite status and stunting occur among healthy children living in similar environment (Fig. 1).

### Early Childhood Appetite and Satiety Tool (ECAST)

ECAST was administered on mothers/caregivers of the study participants monthly for 6 months. Based on previous literature review and findings from focus group discussions, a 27-item interview-based tool ECAST was developed in Bangladesh. Fourteen FGDs were carried out in rural and urban settings for the development of the tool. The hypotheses for development of tool resulted from the themes and coding of FGDs and appetite assessment tools used in western settings [28].

### Intervention package

The enrolled stunted children received an intervention package on site, which included one egg and 150 ml milk (FS), as well as micronutrient supplementation daily (micronutrient powder (MNP)), 6 days a week, for 3 months, PS and routine clinical care for 6 months [26, 27].

The non-stunted children received routine clinical care, MNP, and nutritional counseling but no FS or PS.

#### Food supplementation (FS)

The FS provided an additional 178 kilocalories, 11.1 g protein, and 11.5 g of fat to the daily diet of enrolled subjects. After enrollment, stunted participants received directly observed nutrition intervention for a maximum of 30 min, 6 days a week, for 3 months, at the community nutrition center (CNC) established on site in Mirpur. Non-stunted children received one egg and 150 ml milk at the CNC under direct supervision of trained research assistants once per month for 3 months, to systematically evaluate food intake. On the day of enrollment, the caregivers of all stunted children were provided a demonstration of responsive feeding practices (recognition of the child’s cues of hunger and appetite and counseling regarding the need to encourage the child to self-feed and praise the child for his/her feeding cues). Mothers/caregivers were discouraged from force feeding their children or administering physical punishment/verbal abuse while feeding. They were also advised not to distract the child while feeding at home (e.g., watching television) and were encouraged to attempt repeated feeding. The mother was requested to bring her child to the designated nutrition center daily for the nutrition intervention to avoid food sharing among family members. Freshly boiled egg and milk were provided every day to each participant. Provided food, intake, and leftover were measured using a structured questionnaire under direct supervision of trained research assistants to make sure that participants were having the controlled diets only. They were also provided with phone numbers of field staff to ensure compliances (such as any information based on study or medical assistance) whenever they required.

#### Psychosocial stimulation (PS)

The PS received by stunted children was comprised of parental counseling provided by trained health workers referred to as play leaders [26, 27]. The play leaders were trained by the study investigators to provide play sessions. The sessions were conducted at the CNC, weekly for 1st month, fortnightly for the 2nd and 3rd months and then monthly for the next 3 months. The total number of visits was 11 over a period of 6 months.

##### Play sessions

The play sessions were comprised of a half an hour play session using culturally appropriate home-made toys with child and mother/caregiver on a one-to-one interaction. Examples of some of the toys are plastic rattles using plastic bottles, drums using discarded tins, balls and dolls made of used clothes, stacking bottles using plastic bottles cut into halves, wooden blocks using discarded wood pieces, push along and pull along toys using discarded tins of bigger size with a string or rope. Mothers/caregivers were fully involved in all the activities and were encouraged to give stimulation to their children by themselves at their home at least three to four times a day. A check-list was used during each visit in an attempt to ensure active participation of the mother/caregiver in providing PS to the child at home.

##### Parental counseling

Parental counseling included a half an hour session, with every mother/caregiver and child focused on improving child-rearing practices and enhancing mother-child interaction for optimal child growth and development. During the sessions, the play leader emphasized the importance of play with the child, chatting, singing song/rhymes, showing love, and giving praise to the child. The play leader also demonstrated how to incorporate play into routine activities like feeding, bathing, sleeping, and getting dressed. The mothers/caregivers were highly discouraged from using physical punishment or verbal abuse to control and discipline the child. The meetings were conducted in a participatory design and mothers/caregivers were encouraged to share their views and offer their suggestions.

#### Routine clinical care

During each follow-up visit at the CNC, all children received routine medical care that included: physical examination, MNP (1 gm of MNP contains 42.90 mg of vitamins, 0.150 mg of folic acid, 10 mg of elemental iron, 4.10 mg of zinc, and 0.56, 0.017, 0.09 mg of copper, selenium, and iodine), growth monitoring, treatment of intercurrent illnesses, health and nutrition education (counseling on infant and young child feeding practices, personal hygiene, hand washing), immunizations under Expanded Programme of Immunization schedule and de-worming (albendazole, 200 mg single dose) if not given in the last 6 months. A physician was assigned to carry out routine clinical examination of the children, provide treatment accordingly and refer children to the study hospital (icddr,b) or other nearby hospitals for further management, if required. Health education and growth monitoring were also provided by trained health workers.

## Data collection

After obtaining written informed consent, on enrollment, trained field research assistants collected anthropometric data and interviewed mothers/caregivers at home using a structured questionnaire, which included information on socio-demography (housing, sanitation, parental education, family income, and wealth) and assessed appetite score (ECAST score).

### Appetite score

The children’s appetite status was assessed by trained field staff, on enrollment and monthly for 6 months at CNC using an interview-based tool, the “Early Childhood Appetite and Satiety Tool (ECAST).” A description of the development and validation of this tool has been published previously [28]. The questionnaire was administered to mothers contained 27 items (detailed in Table 1) focused on “signs and cues of appetite” (5 items), “food consumption” (6 items), “food responsiveness” (4 items), “lethargy” (6 items), “emotion and preference” (6 items). The responses were coded as: never equals 1, sometimes equals 2, and often equals 3. The total ECAST scores were obtained by summing up the responses and higher scores indicate good appetite [28].

**Table 1:** Early Childhood Appetite and Satiety Tool (ECAST)-27 items.

Q	Factor	Item	Code
		In the last 24 h…	1 = Never 2 = Sometimes 3 = Often
1	SC	Did your child point for food that she/he want to eat?	
2		Did your child cry or asked for food?	
3		Did your child tug your clothes to let you know that she/he want to eat?	
4		Did your child show any interest in eating food?	
5		Did your child let you know when she/he was hungry?	
6	CO	Did your child eat more than a few spoons, pinches, or balls of food?	1 = Never 2 = Sometimes 3 = Often
7		Did your child finish all the food that was offered?	
8		How was your child’s food intake?	1 = Less than usual 2 = Usual 3 = More than usual
9		Did your child prefer breast milk more than usual food?^a^	1 = Never 2 = Sometimes 3 = Often
10		Did your child eat on his/her normal schedule (e.g., every 2 or 3 h)…?	
11		Did your child get full before his/her meal is finished?	
12	RF	Did you have to use special activities (singing a song, nursery rhymes, playing a game, showing a video) more than normal to help your child eat?^a^	1 = Never 2 = Sometimes 3 = Often
13		Did you have to spend more time than normal to feed your child?^a^	
14		Did you have to force your child to eat because she/he does not eat what you offered?^a^	
15		Did you have to offer foods in order to get your child to eat that you thought were unhealthy?^a^	
16	LE	Did your child show enough interest to eat?	1 = Never, 2 = Sometimes 3 = Often
17		Did you have to offer only liquids/drinks to feed your child?^a^	
18		Did any illnesses like diarrhea or fever keep your child away from eating less than normal?^a^	
19		Did your child take more than 30 min to finish his/her meal?	
20		Did your child vomit after eating?^a^	
21		Did your child eat very quickly?	
22	EP	Did you need to present foods in an attractive way to get your child to eat?^a^ (ex: colorful food and plates, specific plate, present food in an attractive way)	1 = Never 2 = Sometimes 3 = Often
23		Did you have to arrange special foods to get your child to eat?	
24		Was your child interested to eat?	
25		Did your child enjoy his/her meal when praised or encouraged?^a^	
26		Did your child want the same food even after different foods were offered?^a^	
27		Did your child preferred to eat his/her favorite foods	

*SC* sign and cues, *CO* consumption, *EP* emotional preference, *FR* food responsiveness, *LE* lethargy.

^a^Reverse coding for data analysis.

### Anthropometry

Anthropometry (weight and length) measurements were taken monthly for 6 months by trained field staff. Body weight was measured using Seca or Tanita scales (Seca 210, Seca Asia Pacific, 50470 Kuala Lumpur), which have an accuracy of 10 g. Length was measured using a stadiometer length board (Seca 217, Secca,13601 Benson Avenue, CA 91710 Chino, USA) with 1 mm accuracy. Anthropometric measurements were taken routinely at a fixed time, preferably morning with minimal clothing and without shoes.

### Food intake assessment

The stunted children were given 30 min to consume the meal (an egg and 150 ml of milk). The control children were given the same food once a month for 3 months to assess their food intake. Both the duration and mass of food consumption were recorded digital cooking scales (TANITA analog cooking scale 1439, Tokyo, Japan) by trained research assistants. Weight of the leftover food was also recorded.

## Results

A total of 100 children were enrolled (50 stunted and 50 non-stunted) into the study, and 77 children (41 stunted and 36 non-stunted) completed the 6 months follow-up. Demographic and baseline data are presented in Table 2. At enrollment, stunted children had a median age of 14 months and a median LAZ of −2.44, and non-stunted children had a median age and LAZ of 15 months and −1.19, respectively. In both groups, mothers were the primary caregivers, with a median age of 25 years.

**Table 2:** Baseline characteristics of stunted and non-stunted children.

Characteristics	Stunted *n* = 50 (%)	Non-stunted *n* = 50 (%)	*P* value
Age of caregivers (years), median (IQR)	23 (20, 27)	23 (20, 28)	0.449
Type of caregivers: mother	49 (98)	50 (100)	1.000
Grade of schooling			0.626
No education	10 (20)	14 (28)	
Primary	20 (40)	19 (38)	
Secondary and above	20 (40)	17 (34)	
Occupation			0.338
House wife	43 (86)	46 (92)	
Working women	7 (4)	4 (8)	
Age of child (months), median (IQR)	14 (13, 18)	15 (12, 16)	0.667
Length-for-age *z*-score, mean ± SD	−2.44 ± 0.27	−1.19 ± 0.40	<0.001
Sex of the child: female	30 (60)	27 (54)	0.545
H/o exclusive breast feeding	30 (60)	36 (72)	0.205
Appropriate onset of CF	31 (62)	34 (68)	0.529

### Appetite score

**Fig. 2:**
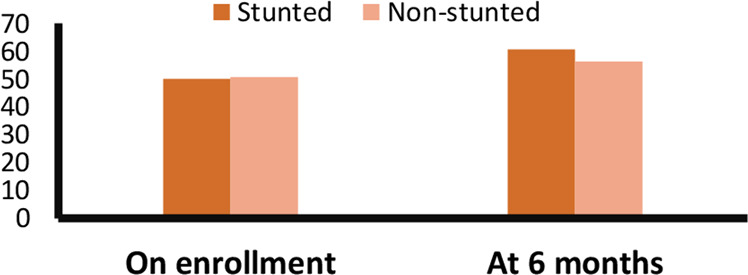
Appetite status of the children on enrollment and after 6 months of intervention. We provided food supplementation 6 days a week for 3 months and measured appetite score monthly. The mean appetite score was 50 in stunted children at enrollment, and increased to 60 after 6 months of intervention whereas the mean appetite score was 51 in non-stunted children at enrollment, and rose to 56 over the same 6-month period.

Measurements of appetite score are presented in Table 3 as “on enrollment,” “at the end of the 3 months,” and “at the end of the 6 months.” The mean appetite score was 50 in stunted children at enrollment, and rose to 60 after 6 months of intervention (Table 2) and increases gradually (Supplementary Table 1). The ten-point difference represents a significant increase from baseline. The mean appetite score was 51 in non-stunted children at enrollment, and rose to 56 over the same 6-month period (Fig. 2 and Supplementary Fig. 1).

**Table 3:** ECAST score among stunted and non-stunted group on enrollment, at 3 and 6 months.

	Stunted			Non-stunted		
	Mean	Upper limit	Lower limit	Mean	Upper limit	Lower limit
On enrollment	49.8	48.6	51.1	51.5	49.9	53.2
At 3 months	56.7	54.8	58.7	54.0	52.2	55.8
At 6 months	60.3	58.2	62.4	56.3	54.3	58.4

### Food consumption

We provided one egg (average weight is maximum 50 g) and 150 mL of milk daily for 90 days to stunted children, and the same food was provided once in a month to measure appetite score and food intake for 3 months among non-stunted children. Children in both groups increased food consumption during the study. Stunted children consumed 40.3 g egg at enrollment which increased significantly to 49.6 g at end line, and 83.8 ml milk at baseline and 138.5 ml milk at end line. Similarly, non-stunted children consumed 40.3 and 50.0 g egg, and 92 and 130 ml milk at baseline and end line accordingly (Table 4 and Supplementary Table 2).

**Table 4:** Changes in food consumption in baseline and end line in stunted children.

Type of food intake	Stunted			Non-stunted		
	Baseline (*n* = 50)	End line (*n* = 41)	*P* value^a^	Baseline (*n* = 50)	End line (*n* = 36)	*P* value^a^
Consumption of egg (g) (mean ± SD)	40.36 ± 16.29	49.63 ± 13.87	0.003	40.35 ± 19.11	50.02 ± 12.75	0.007
Consumption of milk (ml) (mean ± SD)	83.88 ± 43.75	138.58 ± 23.94	0.000	91.98 ± 46.42	129.52 ± 32.99	0.000

^a^Difference shown between baseline and end line by paired *t*-test.

## Discussion

The primary objective of the study was to see the changes in appetite status of stunted children after receiving nutritional intervention. Prior studies have assessed the use of an appetite score based on food consumption, whereas the present study measured appetite using a previously validated interview-based tool [28, 30]. The results of this study suggest that stunted children in Bangladesh receiving a nutrition intervention comprised of FS and PS had measurably increased appetite over a period of 6 months.

A number of studies have shown positive impact of nutrition interventions on nutritional status and child development in malnourished children [25–27]. A prior study of stunted children ages 9–24 months, divided into four groups receiving either FS or PS, both, or none, demonstrated a significant beneficial effect of FS and PS on child development through early adolescence [31].

Any food-based intervention must include nutritious, available and affordable food, and avoid social and cultural taboos [32]. Numerous studies have demonstrated significant increases in linear growth and reduced stunting with milk or eggs. Interestingly, all the studies that intervened with milk were limited to age groups between 6 and 17 years [33–36]. The present study provided both eggs and milk, 6 days a week for 90 days, to children in a younger age group, where stunting is more prevalent [37]. The duration of the FS in this study was similar to that reported in other studies of nutritional intervention in Bangladesh (3 months) [27, 38].

Micronutrient supplementation was included in the nutrition intervention package in this study. The increased appetite score among stunted children could suggest that the lack of appetite at enrollment was a result of specific micronutrient deficiencies in this population. This result is supported by a prior study conducted in Southern Benin, where micronutrient supplementation improved appetite and growth among young children [12].

Positive parent–child communication and activities that encourage creative play and social connection facilitate the development of child’s appetite [39]. A number of studies have shown that children under 3–4 years of age eat primarily in response to appetite or hunger cues, whereas older children’s eating habits are influenced by a variety of environmental and social factors [40, 41]. We provided parenting intervention through PS sessions, which may have helped to increase interest in food intake including understanding of appetite or hunger cues. We calculated appetite scores monthly to determine whether there were subsequent improvements in scores.

We provided FS for 3 months and PS for 6 months to the stunted children. Interestingly, the baseline appetite score and food intake were almost the same in both stunted and non-stunted children in this study. Other studies have also observed similar appetite and food intake patterns in stunted and non-stunted children [31]. At the end of 6 months, stunted children had increased appetite scores. Interestingly, we also found increased food intake over time in the non-stunted children without any nutrition intervention. This may reflect temporal or natural changes in appetite with age or could be a result of micronutrient supplementation. However, micronutrient supplementation alone did not increase appetite among young Beninese children [42]. In our study, we provided food at the community nutrition clinic and stunted children consumed the provided food under direct supervision of field research assistants assuring that no food was shared with other family members.

This study provided a combined intervention package of animal-based foods, micronutrient supplementation, PS, and routine clinical care to stunted children. The study provides an initial assessment of the impact of FS and PS on appetite status using an interview-based tool (ECAST). In this study, stunted children who received CNC-delivered nutrition supplementation and PS exhibited increased appetite over time. Future trials to determine whether such improvements in appetite are also associated with improved nutritional recovery are needed.

## Strengths and limitations

This study had several important strengths. First, the study was conducted in a population where stunting is highly prevalent and interventions to reduce the adverse consequences of stunting are desperately needed. The study used a validated tool to measure appetite and data were collected by well trained staff in a research setting. However, the study also had several limitations. First, the inclusion of a group of non-stunted children was useful as a reference but as these children were not recruited in the same manner and did not receive the same intervention, they cannot be considered a true control group. In addition, the lack of a control group of individuals who did not receive the same intervention makes it impossible to attribute the change in appetite score to the intervention. Future trials will be needed to confirm the effect of such interventions on appetite and subsequent growth.

## Supplementary information

Changes in appetite score between stunted and non-stunted children (27 questions)

Changes in appetite score in baseline (on enrollment) and end line (at the end of 6 month)

ECAST score among stunted and non-stunted group on enrollment, 3 months and at 6 months
