# Supplementary material for: Improvement in appetite among stunted children receiving nutritional intervention in Bangladesh: results from a community-based study
Source: Eur J Clin Nutr. 2021 May 27;75(9):1359–67. doi: 10.1038/s41430-020-00843-9 (PMC8416653; doi:10.1038/s41430-020-00843-9)
Supplement: Supplementary file 1 — Changes in appetite score between stunted and non-stunted children (27 questions) [file 41430_2020_843_MOESM1_ESM.pdf]

Supplementary Table 1: Changes in appetite score between stunted and non stunted children (27 questions)

	**Stunted**		**Non stunted**		**Difference in Difference** *unadjusted*				**Difference in Difference** *Adjusted with occupation, birth order and time of complementary feeding*		
**Month**	**n**	**Mean**	**n**	**Mean**	**Diff**	**DID**	**95% Conf Interval**	**p value**	**DID**	**95% Conf Interval**	**p value**
**On enrollment**	50	49.88	50	50.48	.600						
**Month 01**	46	54.43	48	51.83	2.60	3.201	.570-5.83	.017	3.233	.67-5.78	0.013
**Month 02**	45	54.71	45	52.62	2.08	2.689	-.367-5.41	.053	2.688	.05 5.31	0.045
**Month 03**	42	56.85	38	52.18	4.67	5.273	1.25-9.29	.010	5.418	1.52-9.31	0.004
**Month 04**	40	55.72	34	54.38	1.34	1.943	-1.25-5.13	1.20	2.297	-.90-5.49	0.142
**Month 05**	41	58.85	34	54.35	4.50	5.101	1.75-8.45	.003	5.251	1.9-8.57	0.002
**Month 06**	42	60.34	32	56.53	3.61	4.212	.941-7.482	.012	4.422	1.23-7.61	0.006
