# Supplementary material for: Improvement in appetite among stunted children receiving nutritional intervention in Bangladesh: results from a community-based study
Source: Eur J Clin Nutr. 2021 May 27;75(9):1359–67. doi: 10.1038/s41430-020-00843-9 (PMC8416653; doi:10.1038/s41430-020-00843-9)
Supplement: Supplementary file 2 — Changes in appetite score in baseline (on enrollment) and end line (at the end of 6 month) [file 41430_2020_843_MOESM2_ESM.pdf]

Supplementary Table 2: Changes in appetite score in baseline (on enrollment) and end line (at the end of 6 month)

	**Stunted**		**Non stunted**		**Difference in Difference** *adjusted*				**Difference in Difference** *Adjusted with occupation, birth order and time of complementary feeding*		
**Month**	**n**	**Mean**	**n**	**Mean**	**Diff**	**DID**	**95% Conf Interval**	**p value**	**DID**	**95% Conf Interval**	**p value**
**Baseline**	50	49.88	50	50.48	.600						
**End line**	42	60.34	32	56.53	3.61	4.212	.941-7.482	.012	4.422	1.23-7.61	0.006
