# Supplementary figures and images for: Improvement in appetite among stunted children receiving nutritional intervention in Bangladesh: results from a community-based study
Source: Eur J Clin Nutr. 2021 May 27;75(9):1359–67. doi: 10.1038/s41430-020-00843-9 (PMC8416653; doi:10.1038/s41430-020-00843-9)

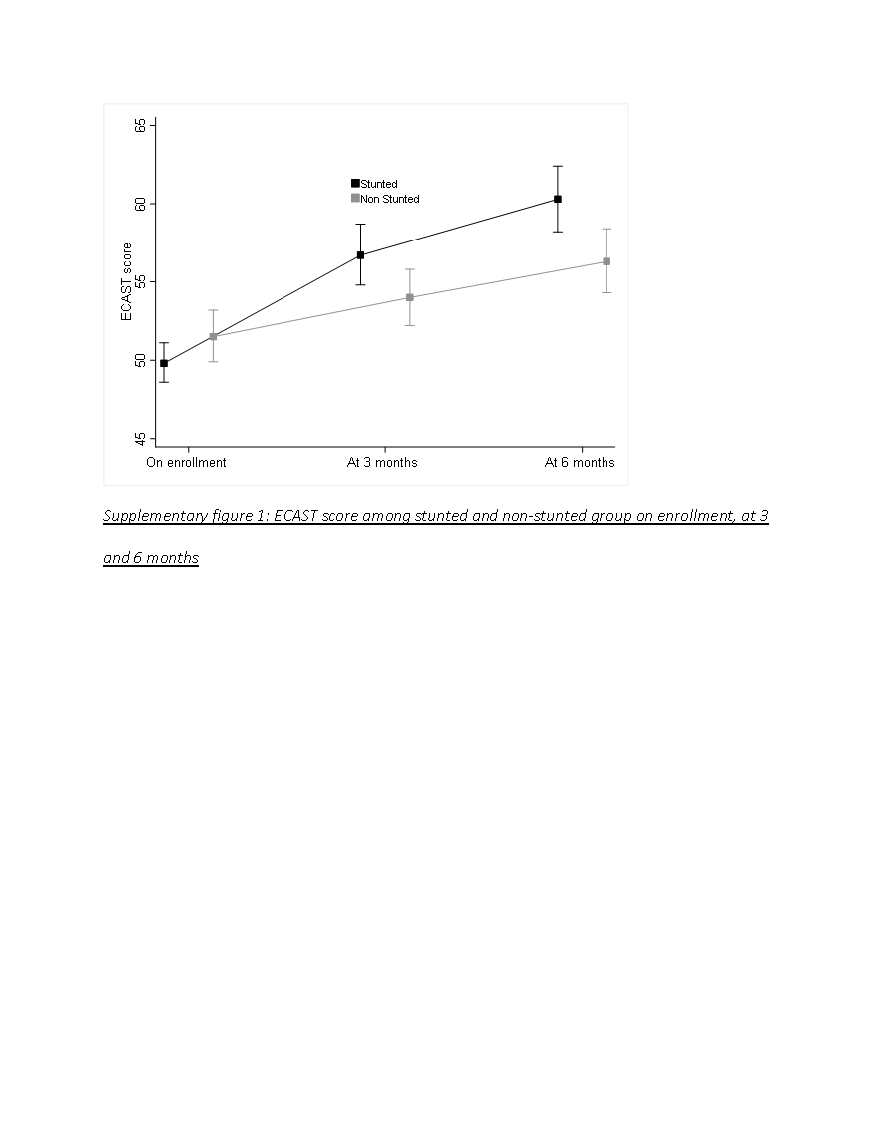

Supplement: Supplementary file 3 — ECAST score among stunted and non-stunted group on enrollment, 3 months and at 6 months [file 41430_2020_843_MOESM3_ESM.tif]
